# Cloning and characterization of farnesyl pyrophosphate synthase from the highly branched isoprenoid producing diatom *Rhizosolenia setigera*

**DOI:** 10.1038/srep10246

**Published:** 2015-05-21

**Authors:** Victor Marco Emmanuel N. Ferriols, Ryoko Yaginuma, Masao Adachi, Kentaro Takada, Shigeki Matsunaga, Shigeru Okada

**Affiliations:** 1 Graduate School of Agricultural and Life Sciences, The University of Tokyo, Japan; 2 Institute of Aquaculture, University of the Philippines Visayas, Philippines; 3 Faculty of Agriculture, Kochi University, Japan

## Abstract

The diatom *Rhizosolenia setigera* Brightwell produces highly branched isoprenoid (HBI) hydrocarbons that are ubiquitously present in marine environments. The hydrocarbon composition of *R. setigera* varies between C_25_ and C_30_ HBIs depending on the life cycle stage with regard to auxosporulation. To better understand how these hydrocarbons are biosynthesized, we characterized the farnesyl pyrophosphate (FPP) synthase (FPPS) enzyme of *R. setigera*. An isolated 1465-bp cDNA clone contained an open reading frame spanning 1299-bp encoding a protein with 432 amino acid residues. Expression of the Rs*FPPS* cDNA coding region in *Escherichia coli* produced a protein that exhibited FPPS activity *in vitro*. A reduction in HBI content from diatoms treated with an FPPS inhibitor, risedronate, suggested that RsFPPS supplies precursors for HBI biosynthesis. Product analysis by gas chromatography-mass spectrometry also revealed that RsFPPS produced small amounts of the *cis*-isomers of geranyl pyrophosphate and FPP, candidate precursors for the *cis*-isomers of HBIs previously characterized. Furthermore, RsFPPS gene expression at various life stages of *R. setigera* in relation to auxosporulation were also analyzed. Herein, we present data on the possible role of RsFPPS in HBI biosynthesis, and it is to our knowledge the first instance that an FPPS was cloned and characterized from a diatom.

Diatoms are one of the most ecologically diverse groups of organisms in today’s oceans with estimates ranging from 30,000 to 100,000 species^1^, all of which play a major role in supporting higher trophic levels and carbon fluxes in marine ecosystems^2^. Despite the extensive diversity of this group, only a handful of species have been found to produce structurally unique C_25_ and C_30_ highly branched isoprenoid (HBI) hydrocarbons^3^. These HBI’s are of great interest given that they have been extensively used as geochemical markers in marine sediments due to their ubiquitous nature^4^, and that some of the C_25_ HBIs have also been found to exhibit cytostatic effects on certain lung cancer cell lines^5^. The unique branched structure of these HBIs also make them a potential form of biofuel through a hydrocracking process similar to botryococcene isoprenoids from the freshwater alga *Botryococcus braunii*^6^,^7^.

Among the handful of diatoms that produce these unique HBIs, *Rhizosolenia setigera* has garnered considerable attention since this species was found to produce a variety of isomers of both C_25_ and C_30_ HBIs depending on the strain, culture conditions, and position in its life cycle^5^,^8^,^9^. Masse and co-workers^10^ established that the HBIs produced by *R. setigera* are mainly produced by the mevalonate (MVA) pathway for isoprenoid biosynthesis through a series of experiments using isotopic labeling techniques. Despite this, the study was only limited to using substrates that were utilized in more upstream processes of the MVA pathway (isotopically labeled acetate, glucose, and CO_2_), and the final steps in the actual synthesis of these hydrocarbons still remain to be elucidated. It has been suggested that the C_25_ and C_30_ HBIs are formed by the attachment of a C_10_ or C_15_ isoprenoid unit respectively at C-6 of another C_15_ isoprenoid unit to produce the unique T branch point observed in their structures^11^,^12^. Farnesyl pyrophosphate (FPP) produced by FPP synthase (FPPS) (EC 2.5.1.10) is the most promising candidate for the C_15_ unit used for the formation of HBIs (Fig. 1).

FPPS is an enzyme that catalyzes the head to tail condensation of two molecules of isopentenyl pyrophosphate (IPP) with dimethylallyl pyrophosphate (DMAPP)^13^ to form FPP. FPP is subsequently converted into a wide range of other natural products such as squalene (through the coupling of two FPP molecules via squalene synthase)^14^, 5-epi-aristolochene (through the cyclization of FPP via 5-epi-aristolochene synthase)^15^, and other volatile sesquiterpenes such as β-farnesene and α-bergamotene^16^. FPP also plays an important role in the prenylation of proteins wherein protein prenyltransferases catalyze the addition of the carbon moeity of FPP to a cysteine residue in the CaaX motif of a target protein, thus facilitating the functional anchoring of these proteins to cell membranes^17^. cDNA cloning of FPPS has already been carried out from a wide range of organisms such as human^18^, rat^19^, yeast^20^, and higher plants^21^,^22^,^23^, and several studies have also highlighted the important regulatory function of this enzyme in producing precursors and substrates for more complex essential metabolites^24^,^25^,^26^.

In the context of *R. setigera*, FPPS presents an interesting research target assuming that a C_15_ isoprenoid unit is used as one of the precursors for the production of C_25_ and C_30_ HBIs. Therefore, the aim of this study was to clone and characterize a potential FPPS cDNA from *R. setigera*, to seek a possible role of the enzyme encoded by this cDNA as the supplier of precursors for HBI biosynthesis, and to determine changes in its gene expression in relation to the position of *R. setigera* in its life cycle. Furthermore, it is to our knowledge the first instance that an FPP synthase was cloned and characterized from the extensively diverse diatoms.

## Results

### Isolation of Rs*FPPS* cDNA

A tentative FPPS cDNA was isolated from *R. setigera* CCMP1694 through a series of bioinformatics and PCR techniques. Using amino acid sequences of known FPP synthases as local BLAST queries and limiting the expectation value [E] of the local BLAST function on BioEdit (V 7.2.3) to 1 × 10^−6^, a single contig was identified from each of the independent *R. setigera* EST databases from Kochi University and the University of Tokyo. One contig corresponded to 1545 bp while the other corresponded to 1474 bp. Both contigs contained a putative open reading frame (ORF) corresponding to 1299 bp (Rs*FPPS*) with 100% identity to each other and encoding 432 amino acids. Using primers designed from these contig sequences, a 1465 bp fragment was amplified by PCR and cloned as the plasmid pBs-Rs*FPPS*1465. This clone contained an ORF of 1299 bases preceded by 21 bases of 5’-UTR and followed by 145 bases of 3’-UTR (Fig. S1).

### Comparison of RsFPPS against FPPS from other organisms

**Figure 2:**
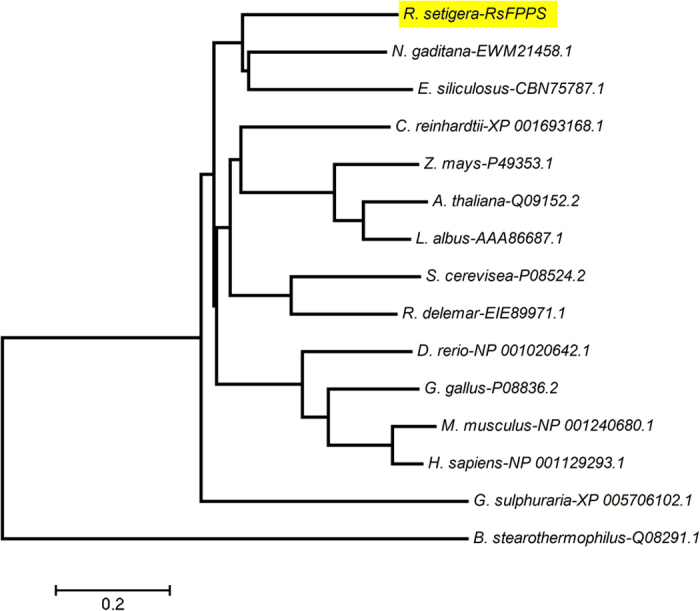
FPPS phylogenetic tree. *R. setigera* FPPS was compared against those from animal, plant, yeast, algae, and bacteria sources using the Neighbor Joining method based on their amino acid sequences. Alphanumeric codes to the right of the binomial names correspond to accession numbers on the NCBI database. The scale bar on the bottom-left is representative of the degree of difference among sequences wherein a distance of 0.2 infers a 20% difference among sequences.

**Figure 3:**
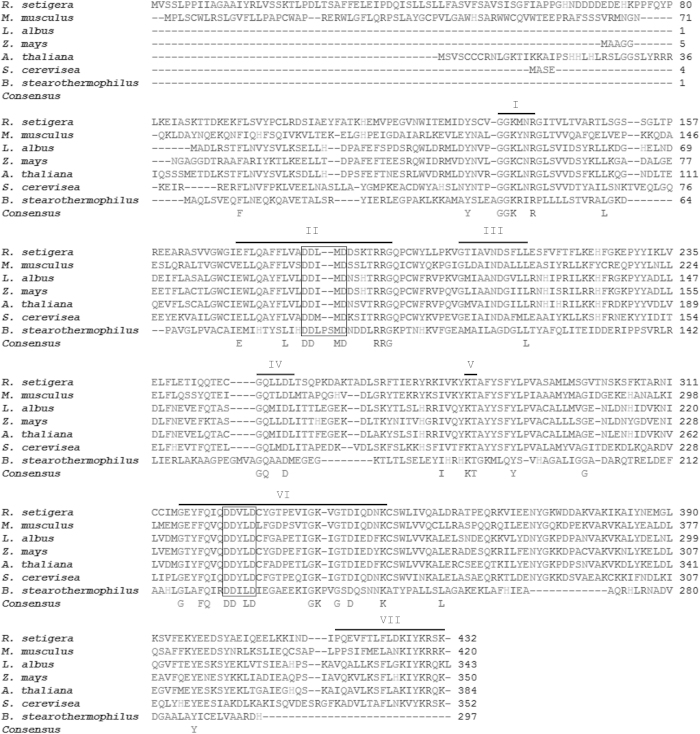
Multiple amino acid sequence alignment of *R. setigera FPPS.* Comparisons were made against those from mouse, yeast, bacteria, and plants. Conserved domains typical of FPP synthases are marked by Roman numerals (I-VII) and a bar above the sequences. The first and second aspartate rich regions in domains II and VI respectively are indicated by boxes.

The predicted molecular weight of *R. setigera* FPPS is 48.94 kDa, which falls within the range for those from mammals (~48 kDa)^27^,^28^, and is relatively larger than those from plants and fungi (39-44 kDa)^20^,^21^,^22^,^29^, and bacteria (~32 kDa)^30^. A BLAST search against the NCBI online protein database showed that the deduced RsFPPS amino acid sequence shared 58% and 55% identity with annotated FPPSs from eustigmatophytes and brown algae, respectively, and 46-51% identity with representative FPP synthase sequences from other organisms (animals, higher plants, green algae, and fungi). Phylogenetic analysis of RsFPPS showed that it was more closely related to algal FPP synthase than to those of animals, higher plants, fungi, or bacteria based on sequence conservation (Fig. 2). Sequence alignment of the putative amino acid sequence of RsFPPS against those of mammals, plants, yeast, and bacteria (Fig. 3) revealed that it contained all conserved amino acid residues necessary for substrate binding and catalytic activity (domains I-VII) typical of other identified FPPSs^31^. Two characteristic aspartate-rich motifs were also present in the RsFPPS sequence (Fig. 3).

### Purification of RsFPPS protein expressed in *E.coli*

The nucleotide sequence corresponding to the open reading frame of Rs*FPPS* (of which the ATG start codon was removed) was inserted into the pET200/D-TOPO expression vector (pET200-Rs*FPPS*1296) and transformed into BL21(DE3) *E. coli* cells. Under the conditions described in Supplementary Methods, appreciable amounts of the recombinant protein were obtained as soluble proteins for Ni-NTA purification.

**Figure 4:**
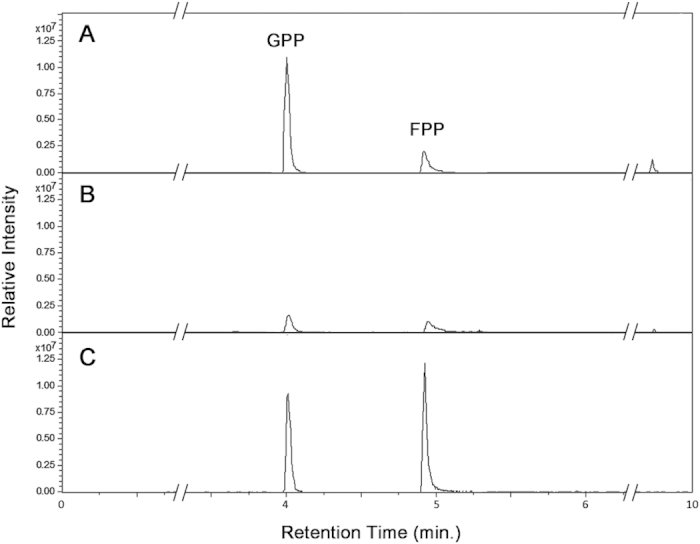
Representative LC/MS extracted ion chromatograms (EIC). **A**) Enzyme reaction with IPP and GPP as substrates, **B**) enzyme reaction with IPP and DMAPP as substrates, **C**) GPP and FPP standards at 100 μM concentrations. GPP and FPP eluted at retention times of 4.0 and 4.9 minutes respectively.

Recombinant RsFPPS, which had an approximate size of 53 kDa due to the inclusion of a 6x-His tag and a linker sequence from the pET200/D-TOPO vector, was isolated to near purity as determined by SDS-PAGE (Fig. S2). The purified protein preparation showed detectable enzyme activity when incubated with IPP and DMAPP or geranyl pyrophosphate (GPP) as seen in the formation of GPP and FPP from liquid chromatography-mass spectrometry (LC/MS) analysis of the reaction mixtures (Fig. 4A,B). Therefore, the *R. setigera* Rs*FPPS* was found to encode for a functional FPPS enzyme.

### Product analysis by LC/MS and enzyme kinetics

An LC/MS assay was established to reproducibly identify FPPS reaction products. LC/MS analysis for the detection and quantification of GPP and FPP standards prepared in sample matrices identical to those used for enzyme assays provided sensitive and reproducible results at concentrations ranging from 0.5 to 250 μM. Sufficient separation of GPP and FPP was achieved with sharp peaks of GPP and FPP at retention times of 4.0 and 4.9 min respectively (Fig. 4C). Identification of RsFPPS reaction products (GPP and FPP) was confirmed by similar retention times (Fig. 4A,B) and mass spectra (Fig. S3) to those of the standards used. This allowed for the determination of steady state enzyme kinetics for RsFPPS, wherein enzyme activity was computed as the amount of FPP molecules formed per min per mg protein.

**Figure 5:**
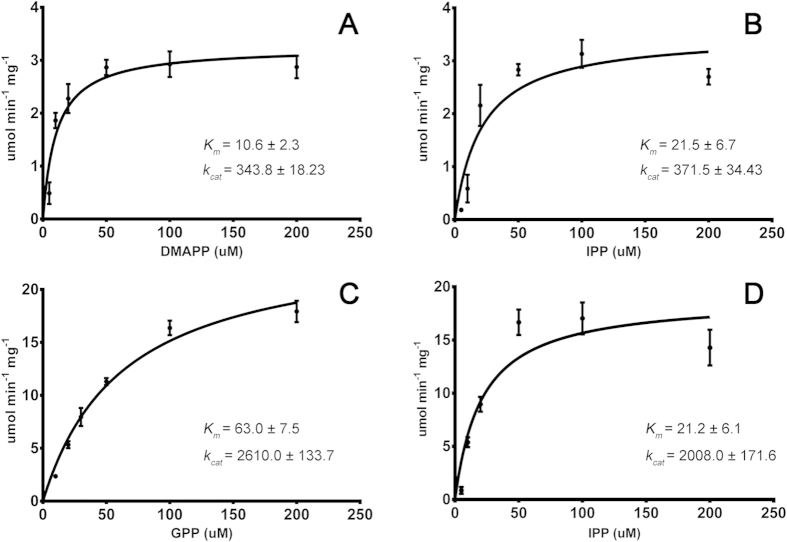
RsFPPS enzyme kinetics. Purified recombinant RsFPPS was subjected to enzyme assays to determine the effect of varying concentrations of the allylic substrates **A**) DMAPP and **C**) GPP on enzyme activity. Parallel reactions using IPP as the counter substrate against **B**) DMAPP (50 μM) and **D**) GPP (100 μM) were also conducted. Inset values (A-D) are the means (±S.D.) of the derived kinetic constants *K*_*m*_ (μM) and *k*_*cat*_(min^−1^). Values for *k*_*cat*_ were calculated using the dimeric form of the enzyme.

Varying concentrations of the substrates IPP, DMAPP, and GPP were used to determine the steady state kinetic constants for recombinant RsFPPS (Fig. 5A-D). For reactions where the IPP concentration was kept constant (50 μM) and DMAPP or GPP were used as counter substrates, the *K*_*m*_ value for DMAPP was 6-fold lower than the derived *K*_*m*_ value for GPP (Fig. 5A,C). In parallel reactions where the DMAPP (50 μM) or GPP (100 μM) concentrations were kept constant and IPP was used as a counter substrate, *K*_*m*_ values for IPP did not differ significantly (Fig. 5B,D). It was noted though, that at higher concentrations of IPP (200 μM), decreases in enzyme activity were observed. Because of this observation, the enzyme-inhibitor constant (*K*_*i*_) for substrate inhibition in these set of reactions was determined, and it revealed that reactions with DMAPP had around two-fold higher *Ki* values for IPP than reactions with GPP (Fig. S5). Conversely, in terms of the turnover rate (*k*_*cat*_) of RsFPPS, reactions where GPP was used as the counter substrate (Fig. 5C,D) showed *k*_*cat*_ values that were 10-fold higher compared to reactions with DMAPP as the counter substrate (Fig. 5A,B), indicating that FPP is more efficiently produced when GPP is used as its allylic substrate.

### Product analysis by gas chromatography/mass spectroscopy (GC/MS)

**Figure 6:**
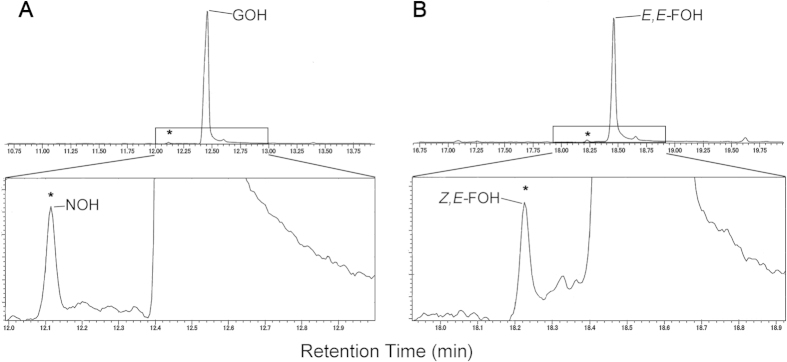
GC/MS total ion chromatogram of enzyme reaction products using IPP and DMAPP as substrates. Upper panels show prominent peaks corresponding to A) geraniol (GOH) and B) *E,E*-farnesol (*E,E*-FOH) with minor peaks for nerol (NOH) and *Z,E*-farnesol (*Z,E*-FOH) marked with asterisks (*). Lower panels are magnified images of the boxed areas in the upper panels. Other minor peaks in the upper panels are either contaminants or artefacts of treatment with alkaline phosphatase as determined in control alkaline phosphatase reactions using GPP or *E,E*-FPP standards.

Products of large scale enzyme assays using IPP and DMAPP as substrates were further verified by GC/MS after converting the generated prenyl pyrophosphates to their corresponding alcohols using alkaline phosphatase. This was done in order to check for possible formation of reaction product *cis*-isomers since separation of GPP and FPP *cis-* and *trans-*isomers was difficult using the described LC/MS conditions. The formation of geraniol (GOH) and *E,E*-farnesol (*E,E-*FOH) was easily detected by GC/MS with peaks appearing at retention times of 12.45 and 18.45 min, respectively (Fig. 6A,B, upper panels; Fig. S4). Interestingly, small amounts of their *cis-* isomers, nerol (NOH) and *Z,E*-farnesol (*Z,E*-FOH), were also detected with retention times of 12.12 and 18.22 min, respectively (Fig. 6A,B, lower panels; Fig. S4). When IPP and GPP were used as substrates, only a small amount of *Z,E*-FOH was detected and no peak corresponding to NOH was observed. The peaks for the *cis*-isomers were not detected when control reactions using GPP and *E,E*-FPP standards were treated with alkaline phosphatase indicating that the expressed RsFPPS produced a small amount of neryl pyrophosphate (NPP) and *Z,E*-FPP as minor products.

### Inhibition of HBI production in diatoms treated with an FPPS-specific inhibitor

**Figure 7:**
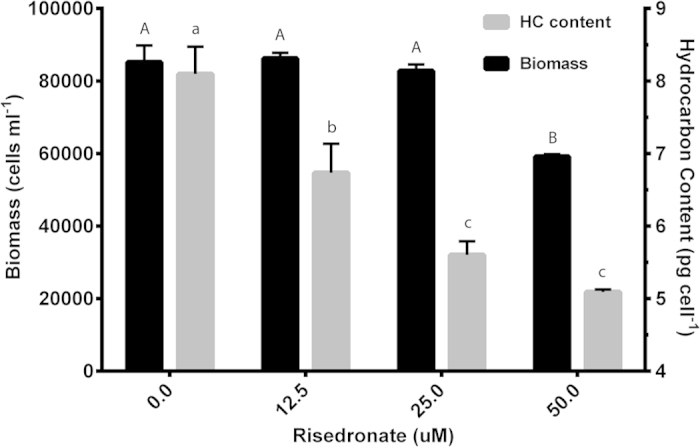
Effect of risedronate on the growth and hydrocarbon content of *R. setigera*. Biomass of *R. setigera* after two-day incubation with risedronate is presented as cells ml^−1^. Total HBI content of *R. setigera* quatified by GC/MS is presented as pg cell^−1^. Values under different letters denote significant differences (p < 0.05) with capital and small letters corresponding to biomass and hydrocarbon content data respectively.

Upon observing that RsFPPS produces potential precursors for HBI biosynthesis, we conducted further experiments to assess how RsFPPS inhibition would affect overall hydrocarbon production. *R. setigera* cultures fifteen cycles after an auxosporulation event (which were mainly producing C_25_ HBIs) were subjected to doses of the FPPS-specific inhibitor risedronate in a range from 0 to 50 μM after a 4-day pre-incubation period (Fig. 7). In terms of overall growth, risedronate did not have a significant effect on the final biomass (cells•ml^−1^) of treated cells for concentrations up to 25 μM. But, overall biomass was reduced by around 25% in treatments with 50 μM risedronate (Fig. 7). Interestingly, total hydrocarbon content (pg•cell^-1^) was reduced in a dose dependent manner with total HBI content decreasing by 16% and 30% at risedronate treatments of 12.5 μM and 25 μM, respectively, compared to the control (Fig. 7). The HBI content of cells treated with 50 μM risedronate were not significantly different from those treated with 25 μM (Fig. 7). This amount can be attributed to HBIs produced during the pre-incubation period prior to the addition of risedronate. These results would suggest that FPPS does contribute C_15_ isoprenoids for HBI production in *R. setigera*.

### Changes in *R. setigera* hydrocarbon composition, cell morphology, and FPPS expression at different life cycle stages

**Figure 8:**
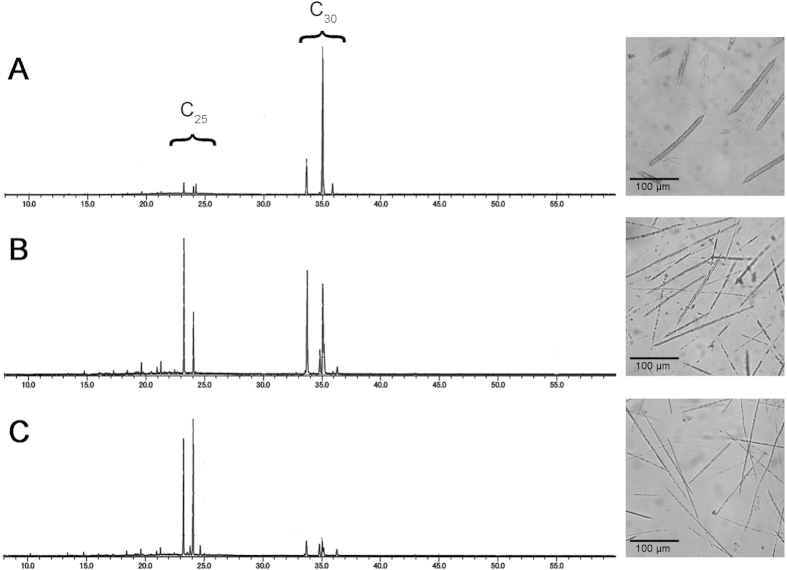
GC/MS total ion chromatograms of hydrocarbon extracts (left panels) and photomicrographs (right panels) of *R. setigera.* **A)** 1^st^ culture cycle upon the onset of auxosporulation, **B**) 10^th^ culture cycle after auxosporulation and **C**) 20^th^ culture cycle after auxosporulation. Ion peaks appearing between 23 to 25 minutes were identified as C_25_ HBIs and peaks appearing between 33 to 36 minutes were identified as C_30_ HBIs based on comparisons of their mass spectra to *R. setigera* HBIs identified in previous studies.

To monitor changes in the HBI composition of *R. setigera*, samples were collected for hydrocarbon analysis by GC/MS at the end of each 15 day culture period from the 1^st^ (Cy1), 10^th^ (Cy10) and 20^th^ (Cy20) culture cycles after auxosporulation as representative samples of *R. setigera* at different stages of its life cycle. Significant changes in HBI composition were notably observed between cycles, wherein a shift from C_30_ HBIs to C_25_ HBIs as the major hydrocarbon component in cells was evident as culture cycles progressed after the auxosporulation event (Fig. 8A-C). Decreasing cell size and increasing cell densities were also noted in the succeeding culture cycles after auxosporulation (Table 1).

Measurement of relative Rs*FPPS* mRNA levels for the corresponding culture cycles mentioned above was analyzed using RT-qPCR techniques with actin (Rs*ACT*) as a reference housekeeping gene for normalization. An increasing trend in Rs*FPPS* mRNA levels was observed in succeeding culture cycles after auxosporulation with a 6-fold increase at Cy20 wherein cell density was highest and cell size was smallest (Table 1). Control experiments with reverse transcriptase omitted and replaced with nuclease free H_2_O during cDNA synthesis gave no detectable signals by qPCR indicating the absence of genomic DNA in RNA preparations.

## Discussion

The use of bioinformatics has become a widely used and reliable tool for the discovery of functional genes. Through these techniques, we were able to screen two sets of *R. setigera* EST databases for a candidate FPPS. Using the information obtained from the EST databases to design gene specific primers, we were able to isolate an Rs*FPPS* cDNA sequence spanning 1465 bp with an ORF of 1299 bp encoding a protein with 432 amino acids and an estimated molecular weight of 48.94 kDa.

The deduced amino acid sequence of Rs*FPPS* cDNA had a high degree of similarity with other known FPP synthases^20^,^21^,^22^,^27^,^29^,^30^. In particular, the highly conserved domains I-VII as outlined by Koyama^31^ were present in the RsFPPS protein. Domains II and VI contained highly conserved aspartate-rich motifs (DDxxD) that were determined to be essential for enzyme substrate binding and catalytic function. The first aspartate-rich motif (FARM) in domain II has been shown to play a role in determination of chain length for the resulting prenyl pyrophosphates through the presence of a conserved Phe residue located five amino acids upstream of the DDxxD motif^32^,^33^. Studies by Tarshis and co-workers^34^ on the crystal structure of recombinant avian FPPS also revealed that the second aspartate-rich motif (SARM) in domain VI is the binding site for the homoallylic substrate IPP. Aside from FPP synthases, both FARM and SARM are also present in other *trans*-isoprenyl pyrophosphate synthases^35^. Enzyme assays carried out using the recombinant *E. coli* expressed and purified RsFPPS protein showed considerable enzyme activity and the formation of both FPP and GPP was observed using both LC/MS and GC/MS analyses. Taken together, these results strengthen our claim that the cDNA isolated in this study represents an *FPPS* gene from *R. setigera* and to our knowledge, is the first instance that an *FPPS* was cloned and characterized from a diatom.

The higher *K*_*m*_ values of RsFPPS for GPP compared to DMAPP as the allylic substrate were similar to those reported for FPPS of the trematode *Schistosoma mansoni*^36^ and FDS-2 from the sagebrush *Artemisia tridentata*^23^ that exhibited preference for DMAPP over GPP. Decreased enzyme activity observed at the highest concentrations of IPP used (200 μM) in the enzyme assays (Fig. 5B,D) point to substrate inhibition typically observed for this enzyme with IPP beyond physiological concentrations^37^. The computed *K*_*i*_ values for these set of reactions revealed that the *K*_*i*_ for IPP was around two times higher in reactions with DMAPP compared to reactions with GPP (Fig. S5). Following the logic presented in previous studies^38^,^39^ that showed substrate inhibition by high concentrations of IPP occurs due to the binding of IPP at the allylic site and that higher concentrations of the allylic substrate are necessary to counter this inhibiting effect, the higher *K*_*i*_ value of IPP for reactions with DMAPP further support the observation that RsFPPS exhibits a preference for DMAPP over GPP as an allylic substrate. In terms of turnover rate (*k*_*cat*_), comparison of reactions using GPP as the allylic substrate showed *k*_*cat*_ values that were on a scale 10-fold higher than those reported for the two FPPSs (FDS-1 and FDS-2) from *A. tridentata*^23^. Higher *k*_*cat*_ values for FPPS have only been reported from the phylogenetically distant bacteria *Bacillus stearothermophilus*^40^ and show an interesting characteristic of RsFPPS (Supplementary Table 1). Given the high catalytic rates of RsFPPS observed in this study compared to those in literature, it stands as a potential candidate for heterologous expression in other model diatoms such as *Phaeodactylum tricornutum*^41^,^42^ which can be used as a platform in relation to efforts to engineer photosynthetic organisms for the production of valuable isoprenoids as biofuels, pharmaceuticals, cosmetics, or other compounds of interest.

GC/MS analysis of the corresponding reaction products after alkaline phosphatase treatment further revealed that RsFPPS also produced a small amount of the *cis*-isomers of GPP and *E,E*-FPP, namely NPP and *Z,E*-FPP, respectively. The formation of these *cis*-isomers tends to occur due to the balancing act that FPPS needs to achieve between proper double bond stereoselectivity of IPP and specific selectivity for IPP against its structural isomer DMAPP at the IPP binding site^37^. Although this phenomenon is not necessarily unique for FPPS as demonstrated in a study by Thulasiram and Poulter^37^, the presence of these *cis*-isomers may provide additional evidence for the hypothetical biosynthetic pathways for the various HBIs produced by *R. setigera* as proposed by Belt *et al.*^12^ and Masse *et al.*^11^. Among the various HBIs from *R. setigera* characterized by Volkman *et al.*^43^ and Rowland *et al.*^9^, structural *cis*- and *trans*- isomers at the T branching point of both C_25_ and C_30_ HBIs have been noted. This leads us to suggest the possibility that both the *cis*- and *trans*-prenyl pyrophosphates produced by RsFPPS could serve as precursors for these HBIs.

To test our hypothesis that RsFPPS provides the precursors necessary for HBI biosynthesis, we conducted *in vivo* inhibition experiments to assess whether FPPS inhibition would affect overall HBI production. Risedronate, a drug commonly used for bone resorption therapy, was determined to specifically target FPPS as its primary mode of action^44^,^45^,^46^. Using this drug, we were able to demonstrate that even at lower concentrations algal growth was not significantly affected, but HBI production was considerably impaired. These observations were similar to those made by Masse and co-workers^10^ using mevinolin, an inhibitor of an upstream enzyme in the MVA pathway, 3-hydroxy-3-methylglutaryl-CoA reductase. Our results therefore point to the possible involvement of RsFPPS in the biosynthesis of HBIs in that inhibition of this enzyme potentially limits the amount of precursors necessary for subsequent HBI formation. Taking this into consideration further supports our argument regarding the involvement of RsFPPS in the proposed hypothetical biosynthetic pathway for HBIs previously put forth.

After demonstrating that RsFPPS produces potential precursors for HBIs, we tried to investigate whether Rs*FPPS* played a regulatory role at the transcriptional level in the biosynthesis of varying types of HBIs produced by *R. setigera* throughout its life cycle. Based on initial observations of C_30_ HBI predominance during the auxosporulation phase of *R. setigera*, we assumed that *R. setigera* would need more C_15_ isoprenoid units produced by RsFPPS during this phase (Cy1) than during the phase when C_25_ HBIs were predominant (Cy20). This assumption was also made based on various hypothetical biosynthetic pathways for HBIs proposed in previous studies^11^,^12^. Measurement of relative amounts of Rs*FPPS* mRNA by RT-qPCR revealed that, contrary to our initial expectation that Rs*FPPS* expression would be higher during auxosporulation due to the abundance of C_30_ HBIs, Rs*FPPS* mRNAs were actually more abundant during cycles when C_25_ HBIs were predominant. This observation could be more in line with a function related to higher growth rates (i.e. faster cell division) rather than the regulation of the types of HBIs between life stages of *R. setigera*. The smaller cell size and higher cell densities observed in later culture cycles (when C_25_ HBIs were predominant) are indicative of faster rates of cell division^47^. In the diatom *Phaeodactylum tricornutum*, increased protein production has been correlated with higher cell densities and faster growth rates^48^. Aside from proteins, sterols, which are derivatives of squalene produced from FPP, are important membrane components in eukaryotes^49^ and sufficient amounts are needed during times of cell division^50^. Studies by Fabris and co-workers^41^ also demonstrated that inhibition of sterol biosynthesis in diatoms was concomitant with reduced growth rates. Given the fact that FPP is located at a branch point in the isoprenoid biosynthetic pathway and is used for various other functions such as protein prenylation and sterol biosynthesis, it could be plausible to assume that the observed increases in Rs*FPPS* mRNA levels are more of a function related to increased growth rates and have little or no regulatory role in terms of the types of hydrocarbons produced by *R. setigera* at various life stages. Furthermore, the relatively small amounts of HBIs produced by *R. setigera* (roughly <1% dry weight) and the nearly similar ratios between their *cis*- and *trans*- isomers^9^ may indicate that it only uses a small fraction of the pool of *E,E*-FPP and GPP to produce the HBIs.

In summary, we have isolated and characterized a cDNA that encodes a functional farnesyl pyrophosphate synthase from the marine diatom *R. setigera*. In our attempts to better understand how this diatom produces unique HBIs, we have presented several lines of evidence that point to the possible contribution of RsFPPS in overall HBI biosynthesis. In terms of the regulation of the types of HBIs produced by *R. setigera* throughout its life cycle though, RsFPPS does not seem to play any significant role and this phenomenon is most likely controlled by a different set of enzymes which can probably use both the *cis*- and *trans*- forms of GPP and FPP as substrates. We are therefore currently undertaking additional studies to discover tentative enzymes from *R. setigera* that can catalyze the final formation of these HBIs.

## Methods

### Algal culture

*Rhizosolenia setigera* CCMP1694 was obtained from the National Center for Marine Algae and Microbiota (Maine, USA). Cultures of *R. setigera* were maintained in 300 ml of f/2 medium^51^ in 500 ml Erlenmeyer flasks under controlled conditions (25 °C, illumination at 250 μmol photons m^−2^ s^−1^, 12:12 light:dark cycle). Culture cycles were carried out over a period of 15 days and after each culture cycle, 50 ml of 15 day-old culture was used to inoculate 250 ml of fresh f/2 medium. All culture cycles were carried out in triplicate. To determine the effect of auxosporulation on the hydrocarbon production and FPPS gene expression levels, cultures were constantly monitored by light microscopy for the onset of auxosporulation as determined by morphological changes (i.e. larger cell diameters). In the initial culture cycle when the onset of auxosporulation was notably observed (denoted as Cycle 1), aliquotes of 100 ml were collected on Whatman GF/C glass filters (GE Healthcare, United Kingdom) by vacuum filtration, freeze-dried for 24 hours, and stored for hydrocarbon extraction and analysis. The rest of the culture was collected on a 20 μm plankton net and the harvested algal cells were snap frozen with liquid nitrogen, and stored at −80 °C for RNA extraction and expression analysis. Similar samplings were carried out in succeding culture cycles.

### Hydrocarbon extraction

The hydrocarbon fraction from *R. setigera* was obtained from the freeze-dried samples collected on GF/C filters by extraction following methods previously reported^52^. The resulting hydrocarbon fraction was subsequently concentrated with a rotary evaporator and a 2.5 μl portion was subjected to GC/MS analysis under conditions described below.

### Rs*FPPS* cDNA cloning

A cDNA library was prepared from RNA extracted from 6 day-old *R. setigera* cells cultured under the same conditions mentioned above. Total RNA was extracted with TRIzol (Invitrogen, Carlsbad, CA) and mRNA was purified using Oligotex^TM^-dT_30_ Super mRNA Purification Kit (Takara, Shiga, Japan). ZAP Express cDNA Synthesis Kit and Gigapack III Gold Cloning Kit (Agilent, Santa Clara, CA) were used for cDNA synthesis and library construction according to the manufacturer’s instructions. The original library composed of 200,000 clones was amplified once in XL 1-Blue MRF’ *E. coli* cells (Agilent).

A putative nucleotide sequence encoding FPPS was mined from two independent *R. setigera* expressed sequence tag (EST) databases by inputting queries of other known sequences for FPPS using the local BLAST function in BioEdit V 7.2.3^53^. From each database, single contigs with high similarity to other FPP synthases were identified. One contig corresponded to 1545 bp while the other contig was 1474 bp long. Both contigs contained a putative open reading frame (ORF) corresponding to 1299 bp (Rs*FPPS*) encoding 432 amino acid residues with 100% identity with each other. To confirm this sequence, the forward primer Rs-*FPPS*-est-F and reverse primer Rs-*FPPS*-est-R (Supplementary Table 2) were designed to amplify a fragment of 1465 bp that included the entire putative coding region and portions of the 5’ and 3’ untranslated regions (UTR) for *FPPS*. PCR amplification was carried out using KOD DNA polymerase (Toyobo Co. Ltd., Osaka, Japan) and the constructed cDNA library as template. The PCR product was purified by agarose gel electrophoresis and ligated to the EcoRV site of the plasmid pBluescript II KS+ (Agilent). The resulting plasmid was designated as pBs-Rs*FPPS*1465 and transformed into XL 1-Blue MRF’ *E. coli* cells (Agilent) by the standard CaCl_2_ protocol. The transformants were plated following protocols for standard blue-white colony selection. Independent positive white colonies were selected to purify plasmids containing the insert for confirmation by sequencing.

### Phylogenetic analysis

The deduced amino acid sequence of RsFPPS was compared to the sequences of other known FPPSs using BLAST^54^. Sequence alignment was carried out using CLUSTAL W2^55^,^56^ against characterized representative sequences from mouse (*Mus musculus*)^27^, yeast (*Saccharomyces cerevisiae*)^20^, bacteria (*Bacillus stearothermophilus*)^30^, and the higher plants *Lupinus albus*^21^, *Arabidopsis thaliana*^22^, and *Zea mays*^29^. A phylogenetic tree was constructed using the Neighbor Joining function on MEGA software version 4.0^57^,^58^,^59^ using the sequences mentioned above along with additional characterized or annotated FPPS sequences from human^28^, avian^34^, piscine^60^, algal^61^,^62^,^63^ and yeast^64^ sources.

### Enzyme assay

Recombinant RsFPPS protein expression and purification are detailed in Supplementary Methods. The purified enzyme’s activity was assayed using methods patterned after those previously described^21^. Unless otherwise stated, reactions were carried out in 1.5 ml Eppendorf tubes in a total volume of 100 μl containing 35 mM Tris-HCl (pH 7.5), 10 mM MgCl_2_, 4 mM dithiothreitol (DTT), 50 μM isopentenyl pyrophosphate (IPP; Sigma-Aldrich, St. Louis, MO), 150 μM dimethylallyl pyrophosphate (DMAPP; Sigma) or geranyl pyrophosphate (GPP; Sigma) as the allylic substrates, and 100 ng of the purified enzyme. Assay mixtures were prepared in bulk without the substrates and the reactions were initiated by substrate addition after sufficient equilibration of the system temperature. For enzyme kinetic analysis, concentrations of the allylic substrates were varied ranging from 5–200 μM. Parallel assays were done with the allylic substrates at a constant concentration (50 μM DMAPP or 100 μM GPP) and IPP as the counter substrate. Reactions were carried out at 30 °C for 10 minutes and stopped by snap freezing the assay tubes in liquid nitrogen. To denature and precipitate the proteins prior to LC/MS analysis, 100 μl of acetonitrile was added to the mixture, vortexed for 10 seconds, and centrifuged at 19,000 *g* for 5 minutes at 4 °C. Detailed analysis and identification of reaction products by GC/MS were carried following the methods of Thulasiram and Poulter^37^.

### Identification and quantification of assay products by LC/MS

Identification of assay products and analysis of enzyme activity were patterned after modified methods of Nagel *et al.*^65^ and Zhang and Poulter^66^. Analysis was carried out on a Shimadzu LC-20AD HPLC system (Shimadzu, Kyoto, Japan) coupled to an AmaZon SL ion trap mass spectrometer (Bruker Daltonics, USA). Separation of reaction products (GPP and FPP) was achieved on an Inertsil ODS-3 column (particle size 3 μm, 4.6 mm ID x 50 mm, GL Sciences, Tokyo, Japan) using a mobile phase consisting of 5 mM NH_4_HCO_3_ as solvent A and 100% acetonitrile as solvent B. A gradient system starting at 0% solvent B, 100% solvent A and increasing to 100% solvent B, 0% solvent A in 8 minutes and a hold at 100% solvent B for an additional 2 minutes was used. A change to 0% solvent B held for 2.5 minutes was done prior to the next injection. Flow rate was at 0.8 ml min^−1^, column temperature set at 40 °C, and injection volume set at 10 μl for all samples and standards. The mass spectrometer was set in negative electrospray ionization mode with ion spray voltage set at −4500 V. For more precise quantification, multiple reaction monitoring was done with transitions of *m/z* 313.4/159.0 for GPP and *m/z* 381.5/159.0 for FPP. To establish calibration curves, FPP and GPP standards (Sigma) were prepared and diluted in the same sample matrix used for enzyme assays. Enzyme activity was computed as the amount of FPP produced per minute per mg of protein. Data analysis was done on DataAnalysis V1.5 and QuantAnalysis V1.3.2 software (Bruker Daltonics). Graphs and statistical analysis for enzyme kinetics were done using GraphPad PRISM software V 6.0 (GraphPad Software Inc., La Jolla, CA, USA).

### GC/MS analysis

Algal hydrocarbon extracts and the corresponding prenyl alcohols obtained by enzymatic dephosphorylation of the enzyme reaction products were analyzed by GC/MS based on methods previously reported^52^. Identification of algal hydrocarbons was based on relative retention times and mass spectra of previously characterized HBIs from *R. setigera* while identification of enzyme reaction products was based on their relative retention times and mass spectra of prenyl alcohols in the mass spectra library on the Shimadzu GCMS LabSolution software V 2.71.

### *In vivo* inhibition of RsFPPS with risedronate

For inhibition studies with risedronate (Tokyo Chemical Industry, Tokyo, Japan), algal cultures fifteen cycles after auxosporulation were used. Using the same conditions mentioned above, a 1L cell culture was allowed to grow for 4 days and subsequently subdivided into 60 ml aliquotes in 100 ml erlenmeyer flasks upon which risedronate was added at concentrations of 0, 12.5, 25, and 50 μM. Each treatment was done in triplicate. The cultures were then allowed to incubate for an additional two days before 50 ml was collected on GF/C glass filters for hydrocarbon analysis.

For quantitative analysis of hydrocarbons in risedronate treated cultures, 10 ng of hexadecane was added to each filter paper as an internal standard prior to extraction and a 2 μl portion was used for GC/MS analysis. Quantification was done by creating a calibration curve using an authentic C_25_ HBI isomer isolated from *R. setigera* as a standard.

### RsFPPS gene expression analysis by RT-qPCR

The relative expression levels of Rs*FPPS* were monitored in cultures of *R. setigera* at various stages in its life cycle. Total RNA was extracted from diatom samples stored at −80 °C as described above. cDNA synthesis for qPCR was done using iScript cDNA Synthesis Kit (Bio-Rad) following the manufacturer’s protocols. qPCR reaction mixtures contained 10 μl of SsoFast EvaGreen Supermix (Bio-Rad), 7 μl of PCR-grade water, 1 μl each of forward and reverse primers (10 μM), and 1 μl of the synthesized cDNA for a total volume of 20 μl. Monitoring of real-time PCR reactions was done on a CFX96 Real-Time System coupled to a C1000 Thermal Cycler (Bio-Rad). Relative expression levels of Rs*FPPS* were normalized against the actin gene (Rs*ACT*) as a reference housekeeping gene^67^. Primers for Rs*FPPS* were designed from the confirmed sequence of plasmid pBs-Rs*FPPS*1465 while primers for Rs*ACT* were designed based on similar contigs mined from the two *R. setigera* EST databases mentioned earlier (Supplementary Table 2). To test for contamination of genomic DNA, extracted RNA was subjected to similar methods mentioned above with the exception that reverse transcriptase was replaced with nuclease free H_2_O in the cDNA synthesis step. The nucleotide sequences for Rs*FPPS* and Rs*ACT* have been submitted to the NCBI GenBank™ database with accession numbers KM360174 and KM360175 respectively.

## Author Contributions

V.M.E.F., R.Y. and K.T. performed the experiments. V.M.E.F. and S.O. designed the study and wrote the manuscript. M.A. contributed the EST database and reviewed the manuscript. K.T. and S.M. provided technical discussions and reviewed the manuscript.

## Additional Information

**How to cite this article**: Ferriols, V. M. E. N. *et al.* Cloning and characterization of farnesyl pyrophosphate synthase from the highly branched isoprenoid producing diatom *Rhizosolenia setigera*. *Sci. Rep.* **5**, 10246; doi: 10.1038/srep10246 (2015).

## Supplementary Material

Supporting Information

## Figures and Tables

**Figure 1:**
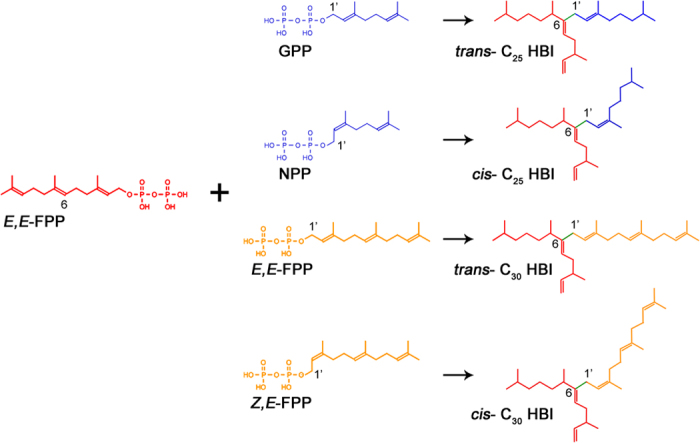
Simplified hypothetical biosynthetic pathway for the formation of representative C_25_ and C_30_ highly branched isoprenoids produced by *R. setigera*. Red structures (*E,E*-FPP) represent the C_15_ isoprenoid unit to which a C_10_ (blue structures, GPP or NPP) or another C_15_ (orange structures, *E,E*-FPP or *Z,E*-FPP) isoprenoid unit attaches at C-6 to produce the *trans-* or *cis-* forms of C_25_ and C_30_ HBIs respectively through 1’-6 coupling. Biosynthetic scheme is patterned after those proposed in previous studies (Masse *et al.*, 2004b; Belt *et al.*, 2006).

**Table 1:** Observed changes in culture cycles of *R. setigera* in relation to auxosporulation in terms of cell density, cell size (diameter), and relative amounts of *RsFPPS* mRNA.

**Cycle**	**Cell Density (cells ml**^**−1**^)	**Cell Diameter (μm)**	**Relative Amounts of RsFPPS mRNA**
Cy1	22,533 ± 1616^a^	12.2 ± 0.6^a^	1.00 ± 0.06^a^
Cy10	80,400 ± 2400^b^	6.1 ± 0.7^b^	2.30 ± 0.26^b^
Cy20	157,733 ± 4105^c^	3.6 ± 0.6^c^	6.55 ± 0.73^c^

Values presented are the mean (± S.D) of 3 replicate culture vessels for cell density, 60 measurements of individual cells for cell diameter, and 4 replicate wells for *RsFPPS* mRNA quantification by qPCR. Values under different superscripts denote significant differences (*p *< 0.05).

## References

[b1] Mann D. G. & Vanormelingen P. An inordinate fondness? The number, distributions, and origins of diatom species. J. Eukaryot. Microbiol. 60, 414–420 (2013). 23710621 10.1111/jeu.12047

[b2] Gao K. *et al.* Rising CO_2_ and increased light exposure synergistically reduce marine primary productivity. Nature Clim. Change 2, 519–523 (2012).

[b3] Sinninghe Damste J. S. *et al.* The rise of rhizosolenid diatoms. Science 304, 584–587 (2004). 15105500 10.1126/science.1096806

[b4] Rowland S. J. & Robson J. N. The widespread occurrence of highly branched acyclic C_20_, C_25_ and C_30_ hydrocarbons in recent sediments and biota - A review. Mar. Environ. Res. 30, 191–216 (1990).

[b5] Rowland S. J., Belt S. T., Masse G., Roussakis C. & Robert J.-M. Effects of temperature on polyunsaturation in cytostatic lipids of *Haslea ostrearia*. Phytochemistry 56, 597–602 (2001). 11281137 10.1016/s0031-9422(00)00434-9

[b6] Melis A. Photosynthesis-to-fuels: from sunlight to hydrogen, isoprene, and botryococcene production. Energy Environ. Sci. 5, 5531–5539 (2012).

[b7] Hillen L. W., Pollard G., Wake L. V. & White N. Hydrocracking of the oils of *Botryococcus braunii* to transport fuels. Biotechnol Bioeng. 24, 193–205 (1982). 18546110 10.1002/bit.260240116

[b8] Belt S. T., Allard W. G., Robert J.-M., Rowland S. J. Effects of auxosporulation on distributions of C_25_ and C_30_ isoprenoid alkenes in *Rhizosolenia setigera*. Phytochemistry 59, 141–148 (2002). 11809448 10.1016/s0031-9422(01)00444-7

[b9] Rowland S. J. *et al.* Factors influencing the distributions of polyunsaturated terpenoids in the diatom, Rhizosolenia setigera. Phytochemistry 58, 717–728 (2001). 10.1016/s0031-9422(01)00318-1 11672736

[b10] Masse G., Belt S. T. & Rowland S. J. & Rohmer, M. Isoprenoid biosynthesis in the diatoms *Rhizosolenia setigera* (Brightwell) and *Haslea ostrearia* (Simonsen). Proc. Natl. Acad. Sci. USA 101, 4413–4418 (2004). 15070732 10.1073/pnas.0400902101 PMC384761

[b11] Masse G., Belt S. T. & Rowland S. J. Biosynthesis of unusual monocyclic alkenes by the diatom, *Rhizosolenia setigera* (Brightwell). Phytochemistry 65, 1101–1106 (2004). 15110690 10.1016/j.phytochem.2004.02.019

[b12] Belt S. T., Masse G., Rowland S. J. & Rohmer M. Highly branched isoprenoid alcohols and epoxides in the diatom *Haslea ostrearia* Simonsen. Org. Geochem. 37, 133–145 (2006).

[b13] Dhar M. K., Koul A. & Kaul S. Farnesyl pyrophosphate synthase: a key enzyme in isoprenoid biosynthetic pathway and potential molecular target for drug development. N. Biotechnol . 30, 114–123 (2013). 22842101 10.1016/j.nbt.2012.07.001

[b14] Pandit J. *et al.* Crystal structure of human squalene synthase. A key enzyme in cholesterol biosynthesis. J. Biol. Chem. 275, 30610–30617 (2000). 10896663 10.1074/jbc.M004132200

[b15] Starks C. M., Back K., Chappell J. & Noel J. P. Structural basis for cyclic terpene biosynthesis by tobacco 5-epi-aristolochene synthase. Science 277, 1815–1820 (1997). 9295271 10.1126/science.277.5333.1815

[b16] Schnee C. *et al.* The products of a single maize sesquiterpene synthase form a volatile defense signal that attracts natural enemies of maize herbivores. Proc. Natl. Acad. Sci. USA 103, 1129–1134 (2006). 16418295 10.1073/pnas.0508027103 PMC1347987

[b17] Clarke S. Protein isoprenylation and methylation at carboxyl-terminal cysteine residues. *Annu. Rev. Biochem*. 61, 355–386 (1992). 1497315 10.1146/annurev.bi.61.070192.002035

[b18] Wilkin D. J., Kutsunai S. Y. & Edwards P. A. Isolation and sequence of the human farnesyl pyrophosphate synthetase cDNA. *J. Biol. Chem*. 265, 4607–4614 (1990). 1968462

[b19] Clarke C. F. *et al.* Molecular cloning and sequence of a cholesterol-repressible enzyme related to prenyltransferase in the isoprene biosynthetic pathway. *Mol. Cell. Biol*. 7, 3138–3146 (1987). 3670308 10.1128/mcb.7.9.3138-3146.1987 PMC367948

[b20] Anderson M. S., Yarger J. G., Burck C. L. & Poulter C. D. Farnesyl diphosphate synthetase. Molecular cloning, sequence, and expression of an essential gene from *Saccharomyces cerevisiae*. *J. Biol. Chem*. 264, 19176–19184 (1989). 2681213

[b21] Attucci S., Aitken S. M., Gulick P. J. & Ibrahim R. K. Farnesyl pyrophosphate synthase from white lupin: molecular cloning, expression, and purification of the expressed protein. *Arch. Biochem. Biophys*. 321 493–500 (1995). 7646076 10.1006/abbi.1995.1422

[b22] Delourme D., Lacroute F. & Karst F. Cloning of an *Arabidopsis thaliana* cDNA coding for farnesyl diphosphate synthase by functional complementation in yeast. *Plant Mol. Biol*. 26, 1867–1873 (1994). 7858223 10.1007/BF00019499

[b23] Hemmerlin A., Rivera S. B., Erickson H. K. & Poulter C. D.Enzymes encoded by the farnesyl diphosphate synthase gene family in the Big Sagebrush *Artemisia tridentata* ssp. spiciformis. *J. Biol. Chem*. 278, 32132–32140 (2003). 12782626 10.1074/jbc.M213045200

[b24] Piłsyk S. *et al.*, Overexpression of erg20 gene encoding farnesyl pyrophosphate synthase has contrasting effects on activity of enzymes of the dolichyl and sterol branches of mevalonate pathway in *Trichoderma reesei*. Gene 544, 114–122 (2014). 24793581 10.1016/j.gene.2014.04.073

[b25] Ling Y., Li Z. -H., Miranda K., Oldfield E. & Moreno S. N. J. The farnesyl-diphosphate/geranylgeranyl-diphosphate synthase of *Toxoplasma gondii* is a bifunctional enzyme and a molecular target of bisphosphonates. *J. Biol. Chem*. 282, 30804–30816 (2007). 17724033 10.1074/jbc.M703178200

[b26] Masferrer A. *et al.* Overexpression of *Arabidopsis thaliana* farnesyl diphosphate synthase (FPS1S) in transgenic *Arabidopsis* induces a cell death/senescence-like response and reduced cytokinin levels. Plant J . 30, 123–132 (2002). 12000449 10.1046/j.1365-313x.2002.01273.x

[b27] Andalibi A. *et al.* Mapping of multiple mouse loci related to the farnesyl pyrophosphate synthase gene. Mammal. Genome 4, 211–219 (1993). 10.1007/BF00417565 8499655

[b28] Sheares B. T. *et al.* Cloning, analysis, and bacterial expression of human farnesyl pyrophosphate synthetase and its regulation in Hep G2 cells. Biochemistry 28, 8129–8135 (1989). 2690933 10.1021/bi00446a025

[b29] Li C. P. & Larkins B. A. Identification of a maize endosperm-specific cDNA encoding farnesyl pyrophosphate synthetase. Gene. 171, 193–196 (1996). 8666271 10.1016/0378-1119(95)00880-2

[b30] Koyama T. *et al.* Thermostable farnesyl diphosphate synthase of *Bacillus stearothermophilus*: molecular cloning, sequence determination, overproduction, and purification. J. Biochem. 113, 355–363 (1993). 8486607 10.1093/oxfordjournals.jbchem.a124051

[b31] Koyama T. Molecular analysis of prenyl chain elongating enzymes. *Biosci. Biotechnol. Biochem*. 63, 1671–1676 (1999). 10586494 10.1271/bbb.63.1671

[b32] Ohnuma S. -I. *et al.* Conversion of product specificity of archaebacterial geranylgeranyl-diphosphate synthase, identification of essential amino acid residues for chain length determination of prenyltransferase reaction. *J. Biol. Chem*. 271, 18831–18837 (1996). 8702542 10.1074/jbc.271.31.18831

[b33] Tarshis L. C., Proteau P. J., Kellogg B. A., Sacchettini J. C. & Poulter C. D. Regulation of product chain length by isoprenyl diphosphate synthases. Proc. Natl. Acad. Sci. USA 93, 15018–15023 (1996). 8986756 10.1073/pnas.93.26.15018 PMC26348

[b34] Tarshis L. C., Yan M., Poulter C. D. & Sacchettini J. C. Crystal structure of recombinant farnesyl diphosphate synthase at 2.6-A resolution. Biochemistry 33, 10871–10877 (1994). 8086404 10.1021/bi00202a004

[b35] Liang P. -H., Ko T. -P. & Wang A. H. -J. Structure, mechanism and function of prenyltransferases. Eur. J. Biochem. 269, 3339–3354 (2002). 12135472 10.1046/j.1432-1033.2002.03014.x

[b36] Ziniel P. D. *et al.* Characterization of potential drug targets farnesyl diphosphate synthase and geranylgeranyl diphosphate synthase in *Schistosoma mansoni*. Antimicrob. Agents Chemother. 57, 5969–5976 (2013). 24041901 10.1128/AAC.00699-13 PMC3837879

[b37] Thulasiram H. V. & Poulter C. D. Farnesyl diphosphate synthase: the art of compromise between substrate selectivity and stereoselectivity. *J. Am. Chem. Soc*. 128, 15819–15823 (2006). 17147392 10.1021/ja065573b PMC2516916

[b38] Laskovics F. M., Krafcik J. M. & Poulter C. D. Prenyltransferase. Kinetic studies of the 1’-4 coupling reaction with avian liver enzyme. *J. Biol. Chem*. 254:9458–9463 (1979). 489545

[b39] Reed B. C., Rilling H. C. Substrate binding of avian liver prenyltransferase. Biochemistry 15:3739–3745 (1976). 182217 10.1021/bi00662a015

[b40] Koyama T. *et al.* Identification of significant residues in the substrate binding site of *Bacillus stearothermophilus* farnesyl diphosphate synthase. Biochemistry 35, 9533–9538 (1996). 8755734 10.1021/bi960137v

[b41] Fabris M. *et al.* Tracking the sterol biosynthesis pathway of the diatom *Phaeodactylum tricornutum*. *New Phytol*. (2014) 10.1111/nph.12917. 24996048

[b42] Apt K. E., Grossman A. R. & Kroth-Pancic P. G. Stable nuclear transformation of the diatom *Phaeodactylum tricornutum*. *Mol. Gen. Genet*. 252, 572–579 (1996). 8914518 10.1007/BF02172403

[b43] Volkman J. K., Barrett S. M. & Dunstan G. A. C_25_ and C_30_ highly branched isoprenoid alkenes in laboratory cultures of two marine diatoms. *Org. Geochem*. 21, 407–413 (1994).

[b44] van Beek E., Pieterman E., Cohen L., Löwik C. & Papapoulos S. Farnesyl pyrophosphate synthase is the molecular target of nitrogen-containing bisphosphonates. Biochem. Biophys. Res. Commun. 264, 108–111 (1999). 10527849 10.1006/bbrc.1999.1499

[b45] Montalvetti A. *et al.* Bisphosphonates are potent inhibitors of *Trypanosoma cruzi* farnesyl pyrophosphate synthase. *J. Biol. Chem*. 276, 33930–33937 (2001). 11435429 10.1074/jbc.M103950200

[b46] Ebetino F. H. *et al.* Molecular interactions of nitrogen-containing bisphosphonates within farnesyl diphosphate synthase. J. Organomet. Chem. 690, 2679–2687 (2005).

[b47] Montagnes D. J. S. & Franklin D. J. Effect of temperature on diatom volume, growth rate, and carbon and nitrogen content: Reconsidering some paradigms. *Limnol. Oceanogr*. 46, 2008–2018 (2001).

[b48] Chrismadha T. & Borowitzka M. A. Effect of cell density and irradiance on growth, proximate composition and eicosapentaenoic acid production of *Phaeodactylum tricornutum* grown in a tubular photobioreactor. J. Appl. Phycol. 6, 67–74 (1994).

[b49] Volkman J. K. Sterols in microorganisms. *Appl. Microbiol. Biotechnol*. 60, 495–506 (2003). 12536248 10.1007/s00253-002-1172-8

[b50] Boutte Y. & Grebe M. Cellular processes relying on sterol function in plants. *Curr. Opin. Plant Biol*. 12, 705–713 (2009). 19846334 10.1016/j.pbi.2009.09.013

[b51] Guillard R. R. L. & Ryther J. H. Studies of marine planktonic diatoms. I. *Cyclotella nana* Hustedt, and *Detonula confervacea* (Cleve) Gran. *Can. J. Microbiol*. 8, 229–239 (1962). 13902807 10.1139/m62-029

[b52] Okada S., Murakami M. & Yamaguchi K. Hydrocarbon composition of newly isolated strains of the green microalga *Botryococcus braunii*. J. Appl. Phycol. 7, 555–559 (1995).

[b53] Hall T. A. BioEdit: a user-friendly biological sequence alignment editor and analysis program for Windows 95/98/NT. Nucleic Acids Symp. Ser. 41, 95–98 (1999).

[b54] Altschul S. F., Gish W., Miller W., Myers E. W. & Lipman D. J. Basic local alignment search tool. J. Mol. Biol. 215, 403–410 (1990). 2231712 10.1016/S0022-2836(05)80360-2

[b55] Larkin M. A. *et al.* Clustal W and Clustal X version 2.0. Bioinformatics 23, 2947–2948 (2007). 17846036 10.1093/bioinformatics/btm404

[b56] Goujon M. *et al.* A new bioinformatics analysis tools framework at EMBL-EBI. Nucleic Acids Res. 38, W695–W699 (2010). 20439314 10.1093/nar/gkq313 PMC2896090

[b57] Saitou N. & Nei M. The neighbor-joining method: a new method for reconstructing phylogenetic trees. Mol. Biol. Evol. 4, 406–425 (1987). 3447015 10.1093/oxfordjournals.molbev.a040454

[b58] Zuckerkandl E. & Pauling L. [Evolutionary divergence and convergence in proteins] *Evolving Genes and Proteins* [ Bryson V. & Vogel H.J. (eds.)] [97–166] (Academic Press, New York, 1965).

[b59] Tamura K., Dudley J., Nei M., Kumar S. MEGA4: Molecular Evolutionary Genetics Analysis (MEGA) software version 4.0. *Mol. Biol. Evol*. 24, 1596–1599 (2007). 17488738 10.1093/molbev/msm092

[b60] Woods I. G. *et al.* The zebrafish gene map defines ancestral vertebrate chromosomes. Genome Res. 15, 1307–1314 (2005). 16109975 10.1101/gr.4134305 PMC1199546

[b61] Merchant S. S. *et al.* The Chlamydomonas genome reveals the evolution of key animal and plant functions. Science 318, 245–250 (2007). 17932292 10.1126/science.1143609 PMC2875087

[b62] Cock J. M. *et al.* The Ectocarpus genome and the independent evolution of multicellularity in brown algae. Nature 465, 617–621 (2010). 20520714 10.1038/nature09016

[b63] Corteggiani Carpinelli E. *et al.* Chromosome scale genome assembly and transcriptome profiling of *Nannochloropsis gaditana* in nitrogen depletion. Mol. Plant 7, 323–335 (2014). 23966634 10.1093/mp/sst120

[b64] Ma L. J. *et al.* Genomic analysis of the basal lineage fungus *Rhizopus oryzae* reveals a whole-genome duplication. PLoS Genet. 5, E1000549 (2009). 19578406 10.1371/journal.pgen.1000549 PMC2699053

[b65] Nagel R., Gershenzon J., & Schmidt A. Nonradioactive assay for detecting isoprenyl diphosphate synthase activity in crude plant extracts using liquid chromatography coupled with tandem mass spectrometry. Anal. Biochem. 422, 33–38 (2012). 22266300 10.1016/j.ab.2011.12.037

[b66] Zhang D. L. & Poulter C. D. Analysis and purification of phosphorylated isoprenoids by reversed-phase HPLC. *Anal. Biochem*. 213, 356–361 (1993). 8238912 10.1006/abio.1993.1432

[b67] Siaut M. *et al.* Molecular toolbox for studying diatom biology in *Phaeodactylum tricornutum*. Gene 406, 23–35 (2007). 17658702 10.1016/j.gene.2007.05.022

