# Supplementary material for: Cloning and characterization of farnesyl pyrophosphate synthase from the highly branched isoprenoid producing diatom Rhizosolenia setigera
Source: Sci Rep. 2015 May 21;5:10246. doi: 10.1038/srep10246 (PMC4440519; doi:10.1038/srep10246)
Supplement: Supporting Information [file srep10246-s1.pdf]

SUPPLEMENTARY INFORMATION

Title: Cloning and characterization of farnesyl pyrophosphate synthase from the highly branched isoprenoid producing diatom Rhizosolenia setigera

Authors: Victor Marco Emmanuel N. Ferriols[1,2]
Ryoko Yaginuma[1]
Masao Adachi[3]
Kentaro Takada[1]
Shigeki Matsunaga[1]
Shigeru Okada[1*]

[1]Graduate School of Agricultural and Life Sciences, The University of Tokyo, Japan
[2]Institute of Aquaculture, University of the Philippines Visayas, Philippines
[3]Faculty of Agriculture, Kochi University, Japan

Correspondence: *Shigeru Okada
Laboratory of Aquatic Natural Products Chemistry
Graduate School of Agricultural and Life Sciences
The University of Tokyo
aokada@mail.ecc.u-tokyo.ac.jp

```
                                                   ATTCGCATTAGACATCCGACC -21
ATGGTTAGCTCTCTCCCACCCATTATTGCTGGTGCTGCAATTTACAGGCTCGTCTCTTCTAAGACACTAC 70
 M  V  S  S  L  P  P  I  I  A  G  A  A  I  Y  R  L  V  S  S  K  T  L

CTGATTTAACTAGTGCCTTTTTCGAGCTAGAGATCCCAGATCAAATCTCGTTGCTCTCCCTCCTTTTCGC 140
P  D  L  T  S  A  F  F  E  L  E  I  P  D  Q  I  S  L  L  S  L  L  F  A

TTCAGTATTTTCTGCTGTTTCTATCTCTGGCTTCATCGCACCTCCTGGACACAATGATGATGATGATGAA 210
  S  V  F  S  A  V  S  I  S  G  F  I  A  P  P  G  H  N  D  D  D  D  E

GATGAACACAAACCACCTTTTCAGTACCCTCTGAAAGAAATCGCATCAAAAACAACAGATAAGGAGAAAT 280
 D  E  H  K  P  P  F  Q  Y  P  L  K  E  I  A  S  K  T  T  D  K  E  K

TTCTGTCAGTTTATCCATGTCTACGTGATTCCATTGCAGAGTACTTCGCAACTAAGCACGAAATGGTTCC 350
F  L  S  V  Y  P  C  L  R  D  S  I  A  E  Y  F  A  T  K  H  E  M  V  P

TGAAGGTGTCAACTGGATCACTGAGATGATCGATTACTCATGTGTTGGAGGAAAGATGAATCGTGGAATC 420
  E  G  V  N  W  I  T  E  M  I  D  Y  S  C  V  G  G  K  M  N  R  G  I

ACCGTCCTCACTGTTGCCCGCACTCTCTCTGGTTCAAGTGGACTCACTCCTAGAGAAGAAGCTCGCGCCA 490
 T  V  L  T  V  A  R  T  L  S  G  S  S  G  L  T  P  R  E  E  A  R  A

GTGTCGTTGGATGGGGAATTGAGTTTCTTCAAGCATTTTTCCTCGTTGCTGACGATTTGATGGATGATTC 560
S  V  V  G  W  G  I  E  F  L  Q  A  F  F  L  V  A  D  D  L  M  D  D  S

TAAGACTCGCCGTGGACAGCCTTGTTGGTACTTATTACCAAAGGTTGGAACAATCGCAGTTAACGATTCT 630
  K  T  R  R  G  Q  P  C  W  Y  L  L  P  K  V  G  T  I  A  V  N  D  S

TTTCTGTTGGAAAGTTTTGTGTTTACTTTCTTGAAAGAACACTTTGGAAAAGAACCTTATTACATCAAAT 700
 F  L  L  E  S  F  V  F  T  F  L  K  E  H  F  G  K  E  P  Y  Y  I  K

TGGTTGAGCTTTTCCTTGAGACCATACAACAAACTGAATGTGGTCAATTACTTGATTTAACAAGTCAACC 770
L  V  E  L  F  L  E  T  I  Q  Q  T  E  C  G  Q  L  L  D  L  T  S  Q  P

AAAAGATGCTAAGACTGCAGACTTATCTCGTTTCACCATTGAAAGATACCGTAAAATTGTCAAGTACAAG 840
  K  D  A  K  T  A  D  L  S  R  F  T  I  E  R  Y  R  K  I  V  K  Y  K

ACTGCGTTCTATTCTTTCTACTTGCCTGTCGCCTCAGCTATGCTCATGAGTGGAGTCACCAATTCTAAGT 910
 T  A  F  Y  S  F  Y  L  P  V  A  S  A  M  L  M  S  G  V  T  N  S  K

CTTTCAAGACTGCTCGTAACATCTGCTGTATTATGGGTGAATATTTCCAGATTCAAGATGATGTCCTTGA 980
S  F  K  T  A  R  N  I  C  C  I  M  G  E  Y  F  Q  I  Q  D  D  V  L  D

TTGTTATGGAACTCCTGAAGTTATTGGTAAAGTTGGTACCGATATCCAGGACAACAAATGCTCATGGTTG 1050
  C  Y  G  T  P  E  V  I  G  K  V  G  T  D  I  Q  D  N  K  C  S  W  L

ATTGTTCAAGCGTTGGATCGTGCAACCCCTGAACAACGTAAAGTGATTGAGGAGAACTATGGAAAATGGG 1120
 I  V  Q  A  L  D  R  A  T  P  E  Q  R  K  V  I  E  E  N  Y  G  K  W

ATGATGCAAAGGTAGCAAAGATTAAGGCAATTTATAATGAAATGGGGTTGAAATCTGTCTTTGAGAAGTA 1190
D  D  A  K  V  A  K  I  K  A  I  Y  N  E  M  G  L  K  S  V  F  E  K  Y

CGAAGAAGATTCATATGCTGAAATCCAAGAGGAGCTAAAGAAAATCAATGATATACCACAAGAAGTTTTC 1260
  E  E  D  S  Y  A  E  I  Q  E  E  L  K  K  I  N  D  I  P  Q  E  V  F

ACTTTGTTCTTGGACAAGATCTACAAACGTTCAAAGTAAAGTAAGCTGTGGAATTTGTGATGCATACAGC 1330
 T  L  F  L  D  K  I  Y  K  R  S  K  *

ATCTGGACCACAATTTGTGTAGGTCCGGAAAACAGAAAAAATTAGTAACCATACTTTATTATCGTGATAC 1400
ATACATGAGGTATCTGGGATACTAATTTTAAATAAACTTCATTC 1444
```

Supplementary Figure S1: Nucleotide and predicted amino acid sequence of *RsFPPS* cDNA from *R. setigera* CCMP1694. The nucleotide sequence includes a 1296-bp ORF, preceded by a 21-bp 5'-UTR, and followed by a 145-bp 3'-UTR. The underlined nucleotide sequences correspond to the sequences used to design primers for initial PCR amplifications using the *R. setigera* cDNA library as template.

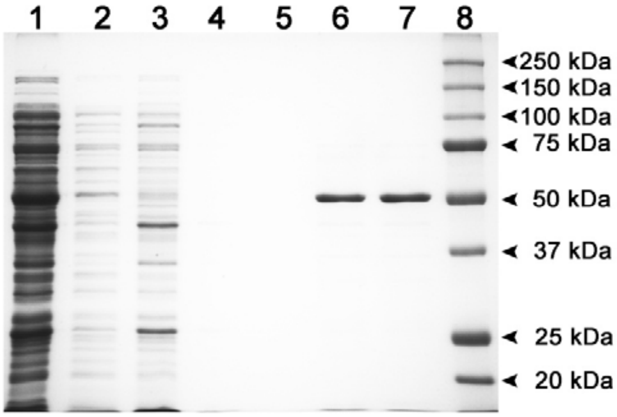

Supplementary Figure S2: SDS-PAGE gel of purified recombinant RsFPPS. *Lane 1*: soluble fraction of cell lysate; *Lane 2*: flow through from Ni-NTA column; *Lane 3*: 1st wash with buffer A; *Lanes 4 and 5*; 2nd and 3rd wash with buffer B; *Lanes 6 and 7*: elution with buffer containing 250 mM imidazole; *Lane 8*: BioRad All Blue Protein Ladder. Gel was stained with Coomasie Briliant Blue.

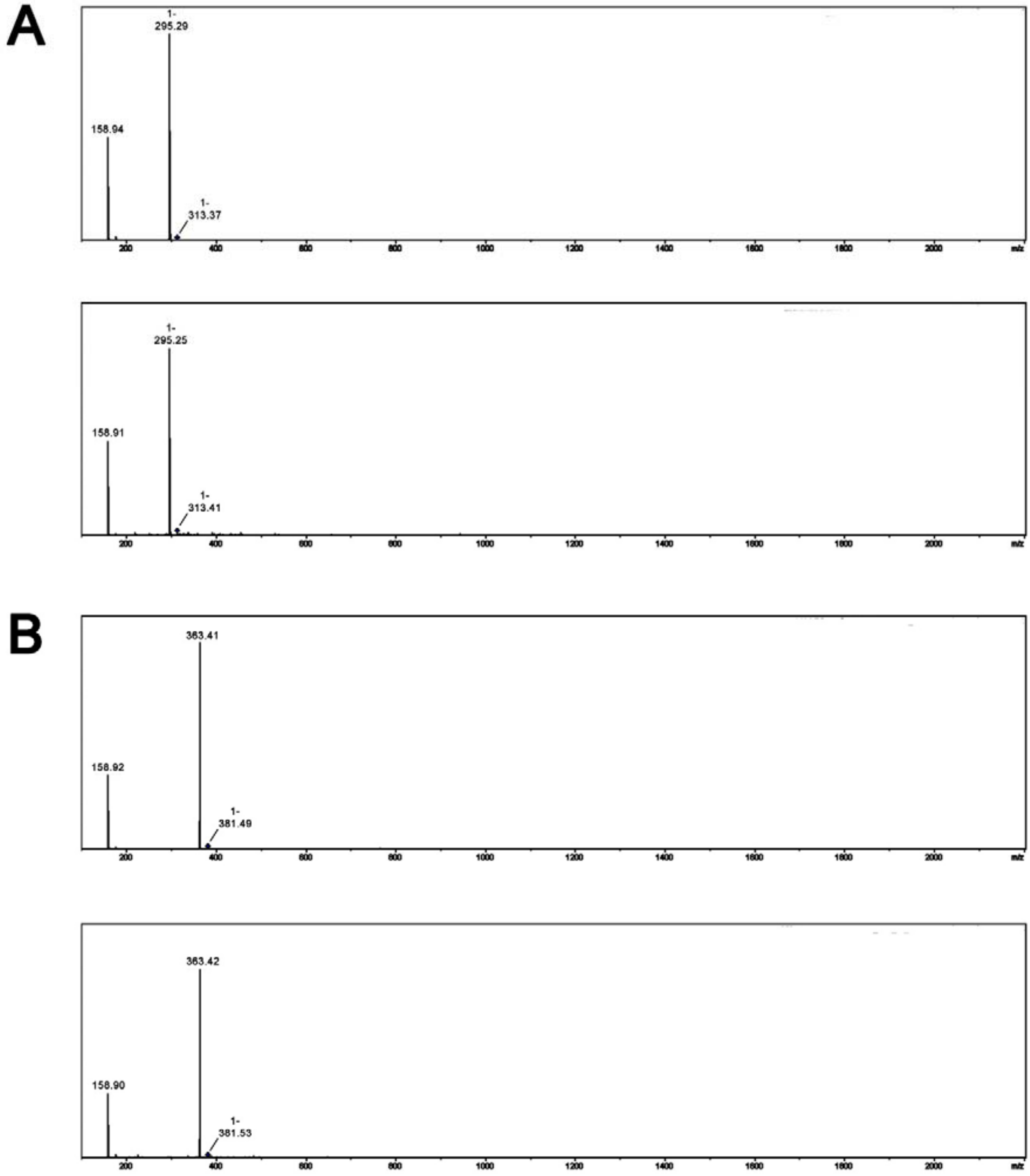

Supplementary Figure S3: Representative LC/MS/MS mass spectra of A) GPP and B) FPP monitored for the quantification of reaction products. Upper and lower panels correspond to standards and RsFPPS reaction products respectively.

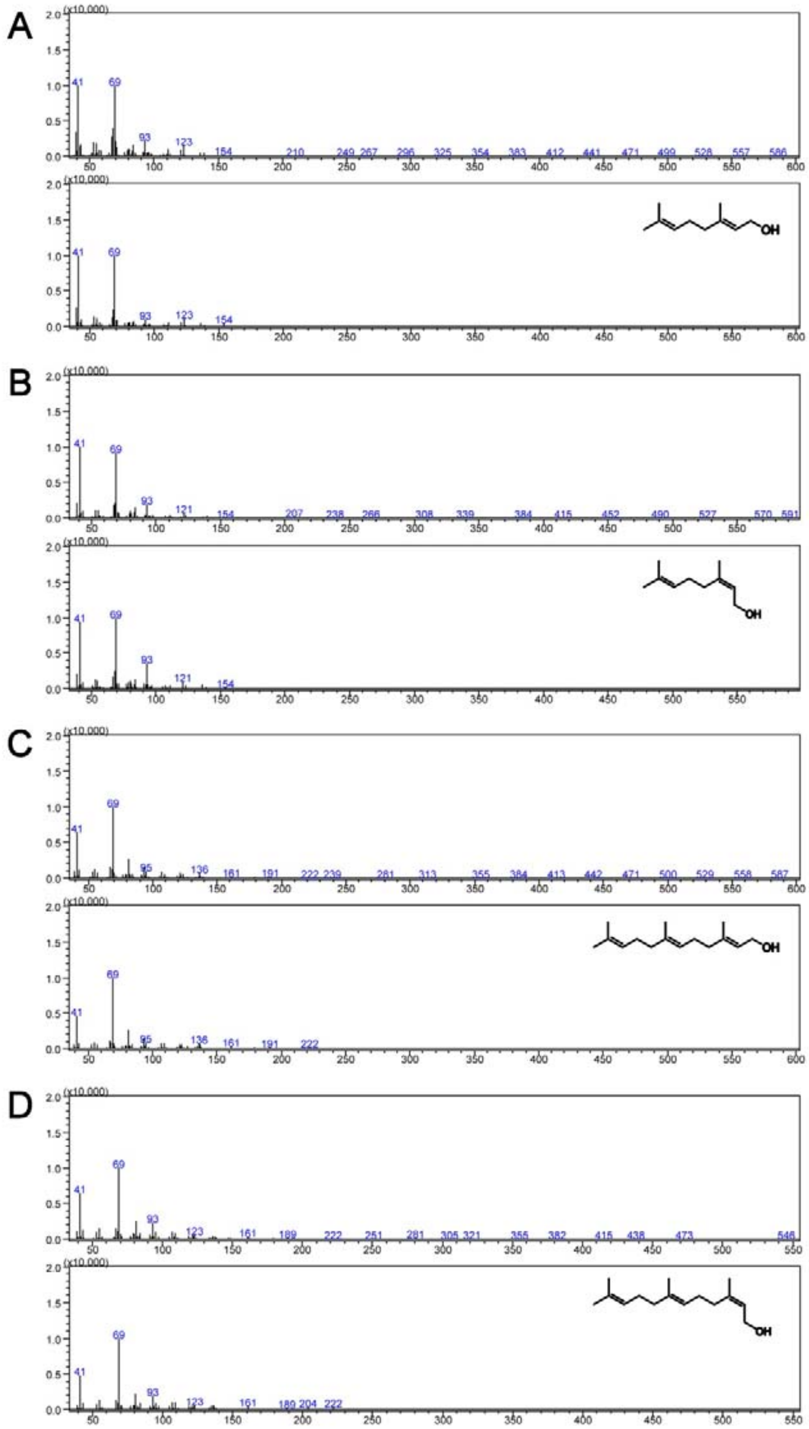

Supplementary Figure S4: Representative GC/MS mass spectra of A) geraniol, B) nerol, C) *E,E*-farnesol, and D) *Z,E*-farnesol. Upper panels correspond to mass spectra of actual FPPS reaction products after alkaline phosphatase treatment while lower panels correspond to mass spectra of prenyl alcohols in the mass spectral library of the Shimadzu GCMS LabSolution software V 2.71.

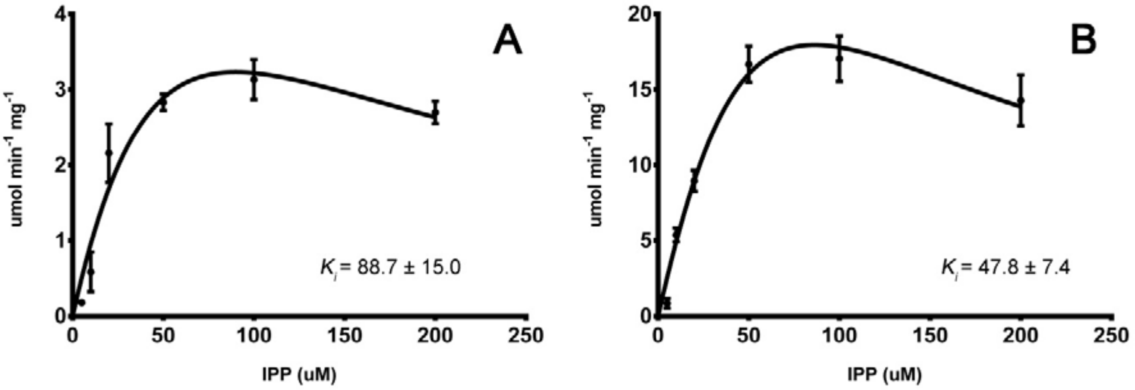

Supplementary Figure S5: Non-linear regression analysis for substrate inhibition to determine K_i values for varying concentrations of IPP as the counter substrate against A) DMAPP (50 µM) and B) GPP (100 µM). Inset values (A and B) are the means (±S.D.) of the kinetic constant K_i (µM) derived from the same dataset used for calculating K_m and k_{cat}.

Supplementary Table 1: Comparison of k_{cat}^{GPP} with FPPS from other studies.

Enzyme	RsFPPS	FDS-1	FDS-2	*B. stearothermophilus* FPPS
k_{cat}^{GPP} (min^{-1})	2008±171.6	159	198	6.60±0.58 x 10^6
Reference	This study	Hemmerlin et al., 2003*	Hemmerlin et al., 2003*	Koyama et al., 1996†

* Reference number 23 in main text

† Reference number 40 in main text

Supplementary Table 2: List of primers used for this study.

Name	Sequence	Use
Rs-*FPPS*-est-F	5'-ATTCGCATTAGACATCCGACC-3'	First PCR
Rs-*FPPS*-est-R	5'-GAATGAAGTTTATTTAAAATTAGTATCCC-3'	First PCR
pET-*FPPS*-FL-F	5'-caccGTTAGCTCTCTCCCAC-3'	Construction of expression vector
pET-*FPPS*-FL-R	5'-TTACTTTGAACGTTTGTAGATCTTGTCC-3'	Construction of expression vector
Rs-*FPPS*-qPCR-F	5'-GTTTTCACTTTGTTCTTGGAC-3'	qPCR
Rs-*FPPS*-qPCR-R	5'-CAAATTGTGGTCCAGATGC-3'	qPCR
Rs-*ACT*-qPCR-F	5'-GTATGTGGATCAGTAAAGAAGAA-3'	qPCR
Rs-*ACT*-qPCR-R	5'-CTAGAAAGCGCAACACATAG-3'	qPCR

Supplementary Methods

Construction of the RsFPPS protein expression vector

Using pBs-Rs*FPPS*1465 as the template, the ORF region for Rs*FPPS* cDNA was amplified by PCR with the primers pET-*FPPS*-FL-F and pET-*FPPS*-FL-R (Supplementary Table 1). The forward primer (pET-*FPPS*-FL-F) was designed to remove the start codon (ATG) in Rs*FPPS* and to enable insertion into the pET200/D-TOPO plasmid (Invitrogen) by the addition of four nucleotides (CACC) at the 5' end. The construct, pET200-Rs*FPPS*1296, was transformed into BL21(DE3) *E. coli* cells (Invitrogen).

Recombinant RsFPPS protein expression and purification

Production of the recombinant RsFPPS was carried out in *E. coli* BL21(DE3) cells harboring the pET200-Rs*FPPS*1296 plasmid grown in LB media with 20 μg ml^{-1} kanamycin . Protein production was induced by the addition of IPTG to a final concentration of 0.05 mM when cell densities were at OD_{600} 0.7~0.9 after incubation at 37 °C. Thirty minutes prior to IPTG induction, sorbitol was added to a final concentration of 600 mM to help with the solubilization of the expressed protein. After induction, cells were grown at 20 °C for 20 hours and harvested by centrifugation at 2,500*g* for 20 minutes at 4 °C. After decanting the supernatant, the resulting pellet was suspended in lysis buffer containing 50 mM NaH_2PO_4 and 300 mM NaCl (pH 8.0) and sonicated with three 5-second bursts using an ultrasonic homogenizer (UD-100, TOMY SEIKO

Co. Ltd., Tokyo, Japan). The lysate was centrifuged at 19,000*g* for 15 minutes at 4 °C and the supernatant was transferred to an equilibrated Ni-NTA column (Qiagen, Hilden, Germany) for purification. Ni-NTA purification was patterned after methods previously described[1,2]. Purification buffers were as follows: washing buffer A (50mM NaH_2PO_4, 300 mM NaCl, 50 mM imidazole, 10 mM ATP, 0.10-0.15 mg ml^{-1} of heat-denatured *E. coli* proteins, and 20% glycerol, pH 8.0), washing buffer B (50mM NaH_2PO_4, 300 mM NaCl, 50 mM imidazole, pH 8.0), and elution buffer (50 mM NaH_2PO_4, 300 mM NaCl, 250 mM imidazole, pH 8.0). To remove the high concentration of imidazole present in the elution buffer, buffer exchange was carried out using a Vivaspin membrane spin column with a molecular cutoff of 10 kDa (Sartorius, Goettingen, Germany). At each step of the purification process, 30 µl aliquots were collected for sodium dodecyl sulfate–polyacrylamide gel electrophoresis (SDS–PAGE) analysis. Purified protein was quantified using the Bradford method (Bio-rad protein assay) with bovine serum albumin as standard[3].

Supplementary Methods References:

1. Guo, L. -W. et al. One-step purification of bacterially expressed recombinant Transducin A-subunit and isotopically labeled PDE6 C-subunit for NMR analysis. *Protein Expr. Purif.* **51**, 187-197 (2007).

2. Rial, D. V. & Ceccarelli, E. A. Removal of DnaK contamination during fusion protein purifications. *Protein Expr. Purif.* **25**, 503-507 (2002).

3. Bradford, M. M. A rapid and sensitive method for the quantitation of microgram quantities of protein utilizing the principle of protein-dye binding. *Anal. Biochem.* **72**, 248-254 (1976).
